# Treatment evolution and improved survival in multiple myeloma in Taiwan

**DOI:** 10.1007/s00277-019-03858-w

**Published:** 2019-12-05

**Authors:** Chao-Hsiun Tang, Hsin-An Hou, Kuan-Chih Huang, Hong Qiu, Yanfang Liu

**Affiliations:** 1 School of Health Care Administration, Taipei Medical University, No.172-1 Keelung Road, Section 2, Taipei, 106 Taiwan; 2 Division of Hematology, Department of Internal Medicine, National Taiwan University Hospital, Zhongzheng Dist, Taipei City, 100 Taiwan; 3 Janssen Research & Development Epidemiology, 319 DunHua South Road, Taipei City, 10669 Taiwan; 4 Global Epidemiology, Janssen Research & Development, 1125 Trenton-Harbourton Road, Titusville, NJ 08560 USA; 5 Global Epidemiology, Janssen Research & Development, 2 Science Park Drive, Singapore, 118222 Singapore

**Keywords:** Asia, Epidemiology, Mortality, Multiple myeloma, Prevalence

## Abstract

The incidence of multiple myeloma (MM) is increasing worldwide, but the rate of increase is greatest in Asia. Few data describe the epidemiology and treatment of MM in Asia. Building on a cohort study from 2007 to 2012 using the Taiwan National Healthcare Insurance Research database, we extended our analysis to estimate the disease burden and treatment patterns of patients with MM in Taiwan through 2015. A further 1664 patients with newly diagnosed MM from 2013 to 2015 (total 4387 patients from 2007 to 2015) were enrolled and followed up until death or end of the observation period (December 31, 2016), whichever occurred first. The age distribution of the 2013–2015 cohort was similar to that for previous years, but there were fewer men (52.1% versus 58.0%), and more patients had renal impairment at diagnosis (19.7% versus 16.4%). From 2007 to 2015, crude annual incidences per 100,000 population of newly diagnosed MM increased from 1.74 to 2.48 and age-adjusted incidences from 1.41 to 1.65. Crude all-cause mortality rates increased over time. Case fatality decreased from 25.5 to 18.3% and median survival increased from 2.10 to 3.12 years. From 2007 to 2015, the percentage of patients receiving first-line therapy with novel agents increased from 0.4 to 89.4%, autologous stem cell transplantation doubled, and chemotherapy use decreased by 81%. Comprehensive national data covering 9 years of follow-up demonstrate continuing change in the disease burden, treatment, and survival of MM in Taiwan. Despite increased use of new treatments, MM remains largely incurable.

**Electronic supplementary material:**

The online version of this article (10.1007/s00277-019-03858-w) contains supplementary material, which is available to authorized users.

## Introduction

Multiple myeloma (MM) is an incurable malignancy of clonal plasma cells that is associated with older age and is more common in men [1]. It is the second most common hematological malignancy after lymphoma, with a global age-standardized incidence in 2016 of 2.1 per 100,000 persons (95% confidence interval [CI] 1.8–2.3) and an age-standardized death rate of 1.5 per 100,000 persons (95% CI 1.3–1.7) [2]. Across the globe, MM incidence rates vary by 10-fold, with the highest rates observed in Europe, North America, and Australia (age-standardized incidence rates 4.6 to 5.8 per 100,000 persons) and the lowest rates in regions of Africa and central Asia (0.4 to 0.9 per 100,000 persons) [2]. The age-standardized incidence rate among African-Americans is twice that among white Americans (9.5 versus 4.1 per 100,000 per year) [3]. The incidence of MM appears to be increasing everywhere, but the highest increase in incident cases from 1990 to 2016 occurred in China, North Korea, and Taiwan (262% increase) [2]. While population growth and an aging population accounted for some of this change, there was a 157% increase in the age-specific incidence rate over time [2]. Few studies have examined how the clinical course of the disease differs between ethnic groups [4]. The factors underlying the lower incidence of MM in Asia and contributing to its increase are not known.

Taiwan has a population close to 24 million. The single-payer National Health Insurance (NHI) system in place since March 1995 provides mandatory health insurance for the entire population of Taiwan. The system provides comprehensive population coverage and accessibility to health services, with low administrative costs [5]. The program is primarily funded by payroll tax premiums and subsidies from government revenue. All administrative and claims data are held centrally (National Health Insurance Research Database, NHIRD) providing a data repository that facilitates research and health policy formulation. We previously used the NHIRD to describe temporal trends in the disease burden, clinical characteristics, and treatment trends of MM in Taiwan from 2007 to 2012 [6]. The age-adjusted incidence of MM increased by 13% over this period, from 1.41 per 100,000 population to 1.59 per 100,000 population (*p* = 0.01). There was a marked change in treatment patterns after the introduction of novel agents (bortezomib, thalidomide, and lenalidomide) to Taiwan, but case fatality in patients with MM remained high (19.4%). In this study, we used the same database to extend the analysis period until 2015. As well as informing on current MM disease trends, treatments, and mortality in Taiwan, these data provide the most up to date information for use in health technology assessments when considering the impact of new treatments for MM.

## Methods

### Data source

The NHIRD population-based claims database is provided by the Taiwan National Health Insurance Administration and maintained by the Health and Welfare Data Science Center, Ministry of Health and Welfare, Executive Yuan, Taiwan [7]. The NHIRD contains information on all medical services in Taiwan. Primary and secondary diagnoses are coded in the International Classification of Diseases, Ninth Revision, Clinical Modification (ICD-9-CM) format, and demographic information, date, and type of service provided (physician services, drugs, prescription, laboratory and imaging examinations, and hospital ward) are recorded. The National Health Insurance Administration verifies the accuracy of the information stored in the database through random reviews of one per 100 ambulatory and one per 20 inpatient claim cases, as well as patient interviews [7–9].

Cases of MM were identified from the Registry of Catastrophic Illness (NHIRD-RCI), a data file within the NHIRD through which insured patients are granted exemption from co-payments for specified major conditions. A catastrophic illness certificate is issued for a list of confirmed diagnoses legislated by the NHI Administration, including cancers. Cytological or pathological reports or evidence supporting the diagnosis of malignancy is required before the certificate is granted.

All personally identifiable information was encrypted to protect patient privacy. Patient consent was not required and the study was granted an exemption from ethical review by the Taipei Medical University-Joint Institutional Review Board.

### Study population

The study population comprised all patients in the NHIRD-RCI who received a catastrophic illness certificate for MM (ICD-9 codes 203.0X or 203.0 or 203) with an initial diagnosis between January 1, 2007 and December 31, 2015. Prevalent cases were defined as all incidents and existing cases of MM captured in the database in each calendar year.

### Outcomes and follow-up

The diagnosis index date was defined as the date of the first MM diagnosis. Patients were followed up from the diagnosis index date until death, end of the follow-up period, or dis-enrollment from the database, whichever occurred first. For the 2007–2012 cohort, the end of follow-up was December 31, 2013. For the 2013–2015 cohort, the end of follow-up was December 31, 2016.

Comorbidities present within 12 months prior to the diagnosis index date were recorded in the subset of patients who had been in the database for at least 1 year. For comorbidities associated with MM, patients were to have had at least three outpatient visit claims or at least one hospitalization claim with a primary or secondary diagnoses of renal injury/renal failure (ICD-9 codes 586), anemia (ICD-9 codes 281, 283, 284, 285, 776), bone fracture (ICD-9 codes 800-829), or pneumonia (ICD-9 code 486). The Charlson Comorbidity Index (CCI) was calculated using ICD-9-CM (Charlson/Deyo).

Three sources were used to capture deaths over the study period: death records in the NHIRD-RCI, discharge status from hospital medical claims, and disenrollment records in the enrollment files of insurance beneficiaries.

Patients who were aged at least 18 years at the diagnosis index date, who had been in the database at least for 12 months prior to the diagnosis index date, who had at least one hospital or outpatient visit for MM after the diagnosis index date, and who had received treatment for their MM were included in the treatment sub-cohort of subjects for assessment of first-line treatment received. In the treatment sub-cohort, patients with a record of any other primary cancer before the MM diagnosis index date and any patients with evidence of a confirmed diagnosis or disease progression to immunoproliferative neoplasms other than MM (ICD codes 203.1X or 203.1 or 203.8X or 203.8) within 2 months after the diagnosis index date were excluded.

The use of chemotherapy, novel agents (bortezomib, thalidomide, and lenalidomide), and steroids for first-line treatment of MM given as an outpatient or inpatient was coded using Reimbursement Codes defined by the NHI administration. Drugs were captured by Anatomical Therapeutic Chemical codes.

### Statistical analysis

Prevalence and incidence rates of MM and mortality among all patients with MM due to any cause were calculated with 95% CIs. The case fatality rate was calculated by dividing the number of the deaths among the total number of MM patients in each calendar year. The all-cause mortality rate was calculated by dividing the number of deaths in patients with MM by the Taiwanese population in each calendar year. Demographic and clinical characteristics were described. Overall survival from the diagnosis index date was calculated using the Kaplan-Meier method. Age standardization used the World Health Organization world population 2000–2025. All analyses were performed using SAS Version 9.4 (Cary, NC, USA).

## Results

### Characteristics of patients with MM in Taiwan

There were 1664 patients in the NHIRD-RCI with an index diagnosis of MM between 2013 and 2015, giving a total for the 2007–2015 period of 4387 patients. The mean age of the 2013–2015 cohort was 67.6 years (standard deviation [SD] 12.0), and 52.1% (867/1664) were men (Table 1). At the time of diagnosis, 37.8% of patients had anemia, 19.7% had renal impairment, 17.9% had a bone fracture, and 15.1% had pneumonia. The mean CCI at diagnosis was 1.9 (SD 2.3).

**Table 1:** Demographic and clinical characteristics of patients with multiple myeloma newly diagnosed from 2007 to 2012 and 2013 to 2015

	2007–2012 *N* = 2723		2013–2015 *N* = 1664	
Variable	Number	Percent	Number	Percent
Gender				
Male	1578	58.0	867	52.1
Female	1145	42.0	797	47.9
Age, mean (SD)	67.6 (12.2)		67.6 (12.0)	
18–29	2	0.1	5	0.3
30–39	40	1.5	16	1.0
40–49	199	7.3	94	5.6
50–59	521	19.1	297	17.8
60–69	686	25.2	494	29.7
70–79	823	30.2	459	27.6
≥ 80	452	16.6	299	18.0
Geographical area				
Taipei	940	34.5	529	31.8
Northern	385	14.1	229	13.8
Central	542	19.9	329	19.8
Southern	396	14.5	260	15.6
Kaohsiung and Pingtung	370	13.6	268	16.1
Eastern	90	3.3	49	2.9
Index year				
2007	383	14.1	-	-
2008	403	14.8	-	-
2009	435	16.0	-	-
2010	479	17.6	-	-
2011	497	18.3	-	-
2012	526	19.3	-	-
2013	-	-	529	31.8
2014	-	-	540	32.5
2015	-	-	595	35.8
Comorbidities associated with MM^a^				
Renal impairment	447	16.4	327	19.7
Anemia	960	35.3	629	37.8
Bone fracture	490	18.0	298	17.9
Pneumonia	471	17.3	251	15.1
Frequency of transplant				
0	2430	89.2	1423	85.5
1	267	9.8	232	13.9
≥ 2	26	1.0	9	0.5
CCI Deyo, mean (SD)	1.8 (2.0)		1.9 (2.3)	
0	834	30.6	563	33.8
1	614	22.5	337	20.3
2	535	19.6	304	18.3
≥ 3	740	27.2	460	27.6

*N* total number of patients in the indicated time period, *n %* number and percentage of patients with the indicated characteristic, *SD* standard deviation, *CCI* Charlson comorbidity index, *MM* multiple myeloma

^a^2592 patients in 2004–2012 and 1661 in 2013–2015 had been in the database for at least 12 months prior to the diagnosis of MM

Compared with the 2007–2012 time period, the 2013–2015 cohort included fewer men (52.1% versus 57.5–58.6% between 2007 and 2012) and a somewhat higher percentage of patients with renal impairment at diagnosis, increasing progressively from 14.4% in 2007–2008 to 19.7% in 2013–2015 (online resource Table S1). No clear temporal trends were observed in MM patients in terms of age, age distribution, or CCI (online resource Table S1).

### Incidence, prevalence, all-cause mortality, and case fatality in patients with MM

**Fig. 1:**
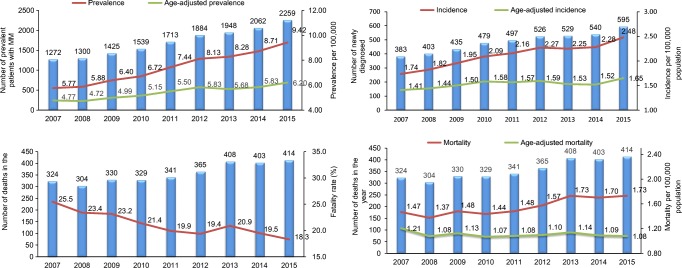
Crude and age-standardized incidence, prevalence, and mortality (all-cause) rates of multiple myeloma in Taiwan from 2007 until 2015

Annual increases in the crude incidence of MM observed between 2007 and 2012 continued through 2015 (Fig. 1), from 1.74 per 100,000 population in 2007 to 2.48 per 100,000 population in 2015. From 2007 to 2015, the age-adjusted incidence rate increased from 1.41 per 100,000 population to 1.65 per 100,000 population, an increase of 17% over the observation period.

The rate of prevalent MM cases increased annually, from 5.77 per 100,000 population in 2007 to 9.42 per 100,000 population in 2015. The age-adjusted prevalence increased from 4.77 per 100,000 population in 2007 to 6.20 per 100,000 population in 2015.

There was no clear trend in crude all-cause mortality rates (death due to any cause in patients with MM divided by the Taiwanese population in each calendar year) apparent between 2007 and 2012 (*p* = 0.1157), but over the longer observation period to 2015, crude mortality increased from a nadir of 1.37 per 100,000 population in 2008 to 1.73 per 100,000 population in 2013 and 2015. Age-standardized all-cause mortality did not change across the 2007–2015 period.

**Fig. 2:**
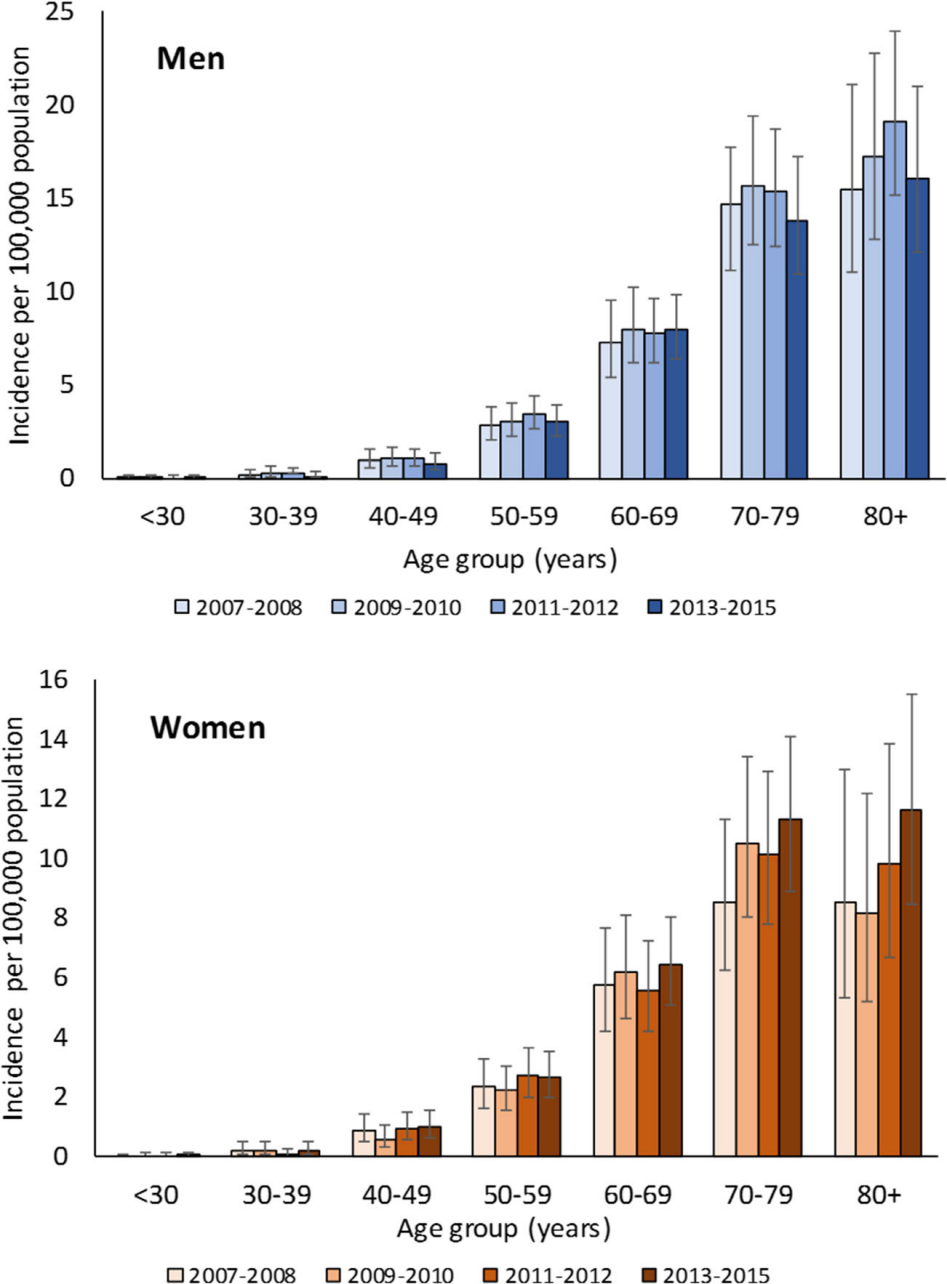
Sex-specific and age-specific incidence of multiple myeloma in Taiwan (age-standardized rates per 100,000 population with 95% confidence interval) 2007–2015. 2007–2012 data from Tang et al [6]. Tabulated data are provided in the online resource Table S2

**Fig. 3:**
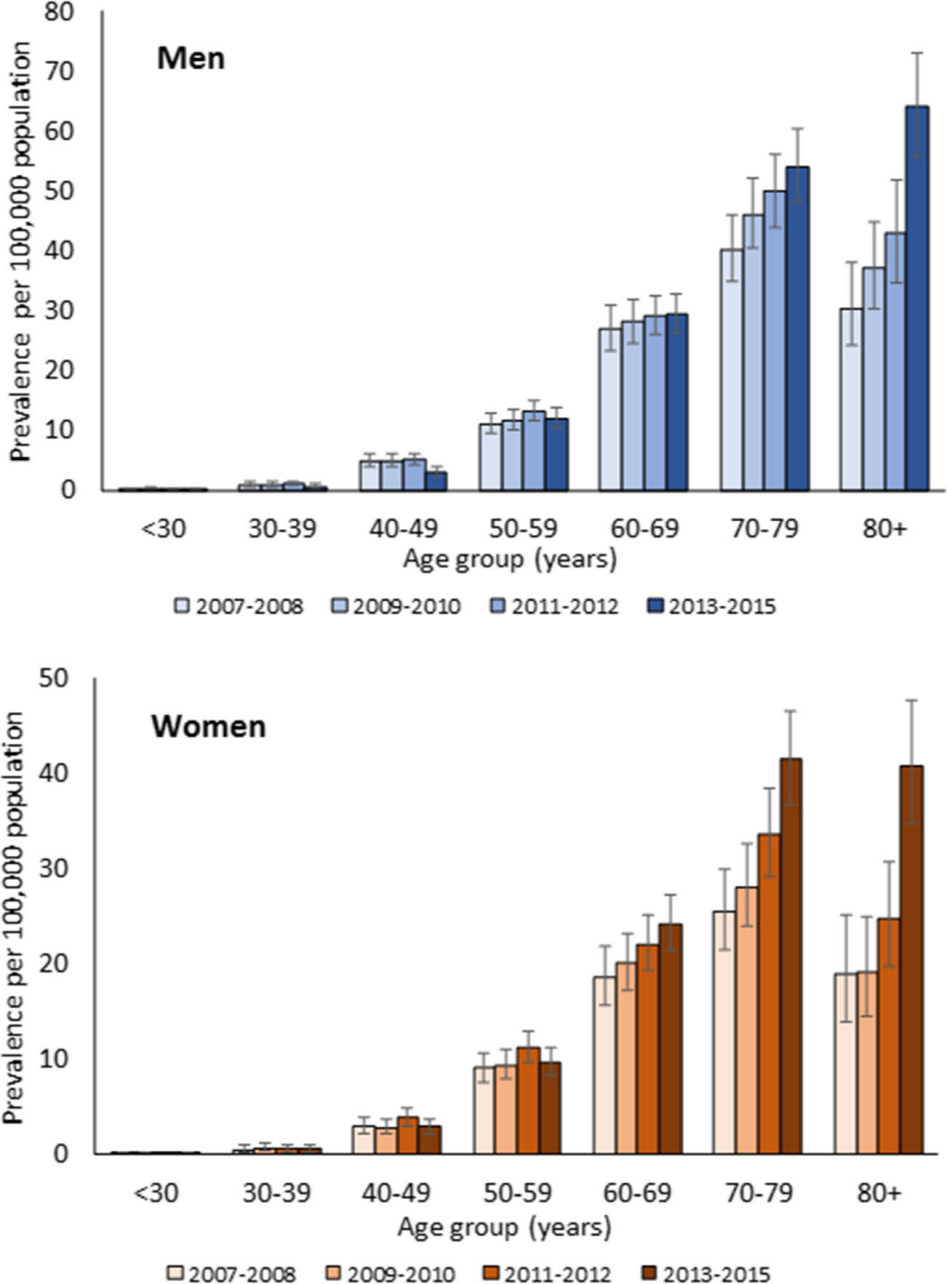
Sex-specific and age-specific prevalence of multiple myeloma in Taiwan (age-standardized rates per 100,000 population with 95% confidence interval) 2007–2015. 2007–2012 data from Tang et al. [6]. Tabulated data are provided in the online resource Table S3

**Fig. 4:**
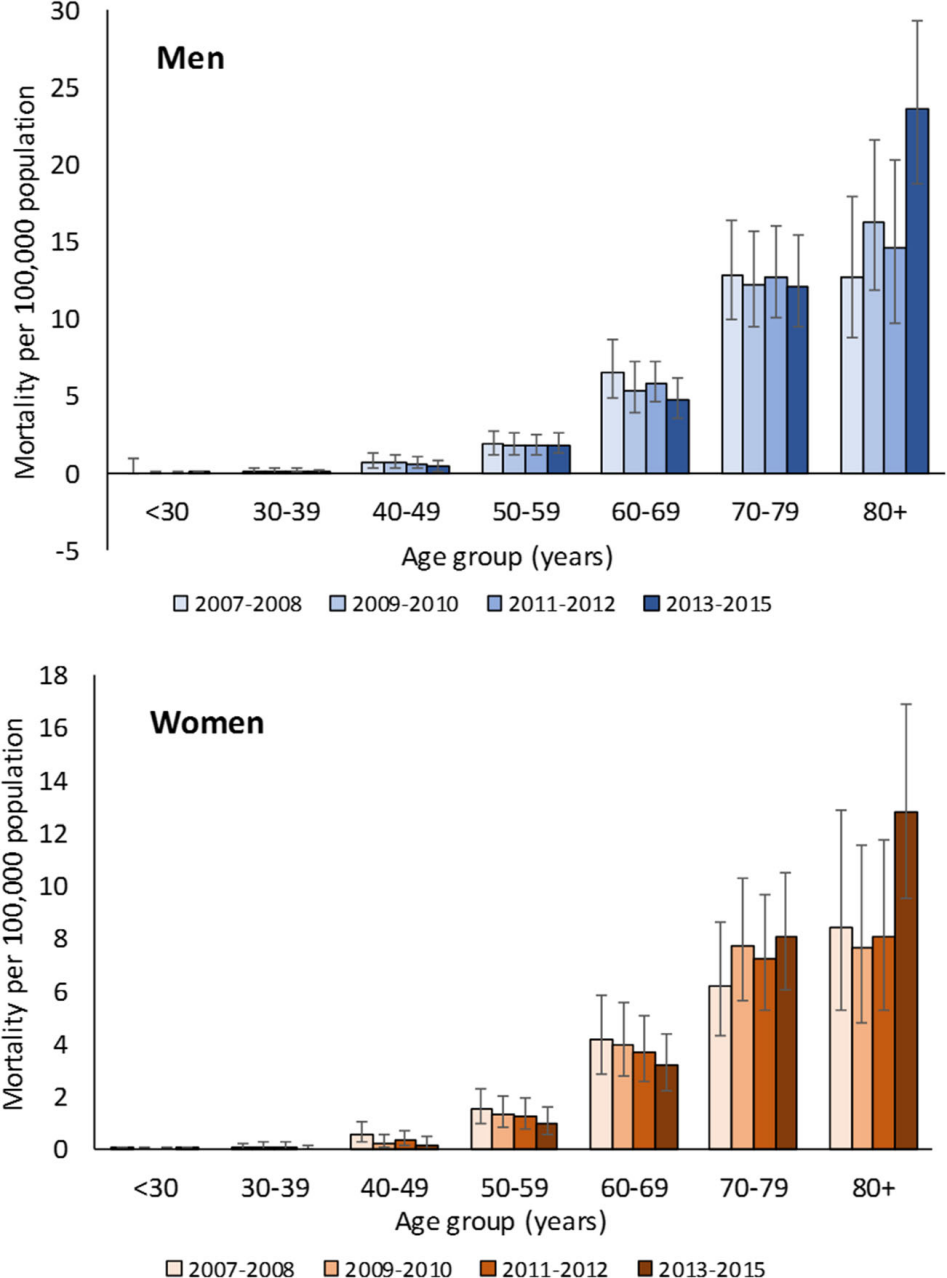
Sex-specific and age-specific mortality (all-cause) of multiple myeloma in Taiwan (age-standardized rates per 100,000 population with 95% confidence interval) 2007–2015. 2007–2012 data from Tang et al. [6]. Tabulated data are provided in the online resource Table S4

Incidence, prevalence, and all-cause mortality in patients with MM increased markedly with age, and the rates were usually higher in men (Figs. 2, 3, and 4). MM incidence peaked in those aged 80 years and older at 16.06 per 100,000 population in men and 11.6 per 100,000 population in women. MM prevalence reached 63.98 per 100,000 population in men aged 80 years and older, versus 40.81 per 100,000 population in women of the same age. The all-cause mortality rate was 23.59 per 100,000 population in men and 12.82 per 100,000 population in women aged 80 years and older.

Compared to the 2007–2012 period, the prevalence of MM in 2013–2015 increased markedly in patients aged 70 years and older (Fig. 3). The overall mortality rate among patients with MM also increased in 2013–2015 compared to previous years; in men from 1.75 per 100,000 population in 2007–2008 to 2.13 per 100,000 population in 2013–2015 and in women from 1.10 per 100,000 population to 1.42 per 100,000 population, respectively (Fig. 4). However, the mortality rate decreased in both sexes for those aged 40–49 years, 50–59 years, and 60–69 years.

The case fatality rate (number of the deaths due to any cause among the total number of MM patients in each calendar year) decreased annually from 25.5% in 2007 to 18.3% in 2015.

### Overall survival

Median survival time was calculated for patients with newly diagnosed MM in 2007–2009 (followed up until December 31, 2013), 2010–2012 (followed up until December 31, 2013), and 2013–2015 (followed up until December 31, 2016), corresponding to the pre-novel agent period, the transition period and post-novel agent periods, respectively. Median OS for all patients increased from 2.10 years in 2007–2009 to 2.60 years in 2010–2012 and 3.12 years in 2013–2015. One-, 2-, and 3-year survival probability also increased over the three time periods (Table 2).

**Table 2:** Median overall survival and survival probability in patients with multiple myeloma in Taiwan, 2007–2009, 2010–2012, and 2013–2015

	2007–2009	2010–2012	2013–2015
Median survival time (years)	2.10	2.60	3.12
Survival probability			
1 year	0.70	0.73	0.75
2 years	0.51	0.57	0.62
3 years	0.41	0.45	0.51
5 years	0.27	-	-

Patients newly diagnosed with MM between 2007 and 2012 were followed by until December 31, 2013). Patients newly diagnosed between 2013 and 2015 were followed up until December 31, 2016

### Evolving patterns of drug use for first-line treatment of MM

**Fig. 5:**
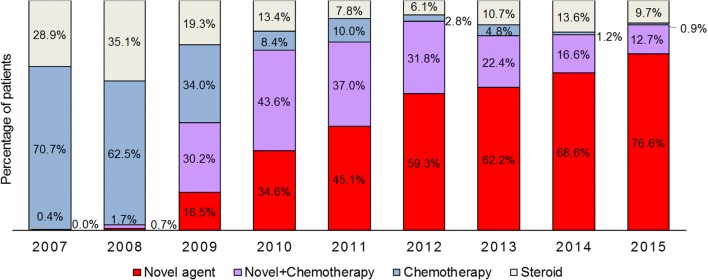
Trends in first-line treatment regimens among patients with multiple myeloma: 1969 patients in 2007 to 2012 and 1576 patients in 2013 to 2015. Novel agents = bortezomib and thalidomide. Tabulated data are provided in the online resource Table S5

The first-line treatment regimen was examined for 1969 patients in the 2007–2012 treatment sub-cohort and for 1664 patients in the 2013–2015 sub-cohort. The use of chemotherapy alone for the first-line treatment of MM virtually ceased over the observation period, from 70.7% of patients in 2007 to 0.9% in 2015 (Fig. 5). Novel agents used alone or with chemotherapy were used increasingly from 2009, such that by 2015, 89.4% of all patients with newly diagnosed MM received first-line therapy with a novel agent.

The percentage of patients who received autologous stem cell transplantation increased from 7.9% in 2007–2008 to 14.4% in 2015 (online resource Table S1).

## Discussion

The results of this extended analysis build on our previous study and confirm a continued increase in the disease burden due to MM in Taiwan since 2007 that as yet shows no sign of plateauing. The age-standardized incidence of MM in Taiwan increased by 17% between 2007 and 2015. Increased case numbers coupled with a 28% decrease in fatality and improved survival resulted in an increase in median survival time by 1 year (from 2.10 to 3.12 years).

We observed an increase in crude all-cause mortality rates (but not age-adjusted all-cause mortality) over time, driven by an increase in patients aged 80 years or more between 2013 and 2015 compared to the preceding years. This likely reflects a combination of increased incidence rates and improved survival in this age group, reflected by the marked increases in MM prevalence among older adults over time.

Thalidomide received re-imbursement for first-line therapy of MM in July 2009. Bortezomib became available in 2007 and received re-imbursement for first-line therapy of MM in June 2012 [10]. Lenalidomide is approved for use in patients with treatment failure of first-line therapy in Taiwan. Since 2007, the first-line treatment of MM has changed dramatically; almost all patients (89.4%) received novel agents alone or combined with chemotherapy in 2015, and the rate of transplantation almost doubled. Over the same period, OS increased and while our study was not designed to show a causal association between treatment use and survival, the increase in OS is consistent with clinical benefit, including increased survival, associated with the use of novel agents and transplantation as demonstrated in clinical trials and epidemiological studies [11, 12].

Although OS has improved in patients with MM in Taiwan, survival rates remain below those observed in other Western countries and other industrialized Asian countries. We linked patient data from the NHIRD concerning first diagnosis of MM with the death registry in Taiwan which enabled us to capture complete information about MM treatments given as inpatients or outpatients, as well as drug access and deaths wherever they occurred in Taiwan. Therefore, we are confident that the estimates of OS in our study are accurate.

A review of MM in 7 countries in Asia concluded that the cytogenetic profiles of patients included in the analysis showed trends similar to the Western literature [13]. However, when these patients were compared with patients with MM from Latin America, it was observed that Asian patients tended be older, to have more advanced disease stages and shorter median OS than those in Latin America [14]. Of note, there was no difference in OS between the regions for transplanted patients, suggesting that different rates of ASCT use influence OS estimates in different countries.

In other industrialized countries in Asia, OS estimates of patients include 47 months (7 countries combined) [1], 60.6 months in Japan [3], and 48.3 months in Korea, versus 37.4 months (3.12 years) in our study. Some of the observed differences could relate to data source and study design. For example, we were able to identify all deaths and avoided loss-to-follow of patients which can occur in hospital-based studies and which can falsely elevate OS estimates. Of note, OS in a study in Korea that also used a national database was 48.3 months. However, 42.5% of patients less than 65 years of age received ASCT in Korea, versus 14.4% in our study.

While we cannot rule out that underlying differences in MM biology contribute to lower survival in Taiwan, the available evidence does not support clinico-pathological differences between MM in Taiwan versus other parts of Asia where survival is higher. The low OS rate observed in our study is likely to be multi-factorial, possibly related to differences in the availability of a range of novel agents, with fewer options currently available in Taiwan than in other countries, as well as the low rate of ASCT in Taiwan.

We observed that all-cause mortality decreased in age groups up to age 70 years but increased in patients aged 70 years and older at the time of diagnosis. Older age is associated with poorer survival from MM due to combinations of frailty, elevated beta-2-microglobulin, comorbidities, increased risk of toxicity from treatment, and general recommendations not to provide ASCT to patients over the age of 65 years [15]. This maxim is being challenged as evidence accumulates that elderly patients can benefit from ASCT and have survival and toxicity profiles that do not differ markedly from younger patients [16, 17]. Kumar et al. (2014) reported improvements in OS in patients with MM, with increases exclusively observed in the 65 years and older age group [11]. This prospective study was conducted at a single institution in which 21% of patients over 65 years of age received ASCT. However, the findings should be interpreted cautiously because the impact of patient loss to follow-up, which can falsely elevate estimations of OS, is unknown. The results of Kumar et al. also contrast with findings from a population-based study in the United States using SEER data that showed no improvement in 10-year relative survival rates from 1993 until 2012 among patients aged 75 years and older [18]. Treatment options for older patients with MM can be improved by early use of novel agents and ASCT. In Taiwan, the observation that mortality in older patients increased over time is concerning and warrants discussion among the medical community on how outcomes in older patients can be improved.

The incidence of MM is increasing globally, most rapidly in Taiwan, China, and Korea [2]. Aging populations and population growth do not fully explain the increase, and other predisposing factors or exposures remain to be fully identified [2]. Exposure to farm work, printing and cleaning occupations appear to have some association with an increased risk of MM [19, 20]. Some studies have identified numerous predisposing genetic factors for MM [21–23]. However, as yet, the role of these exposures and the distribution of genetic susceptibility among different population groups have not been evaluated.

Strengths of our study include the use of the most up to date national data from the NHIRD, a comprehensive data source that captures inpatient and outpatient healthcare-related events for all residents in Taiwan, identification and confirmation of deaths from three sources, and an extended follow-up period over 9 years. The study observation period encompassed the dates when novel agents became available for first-line treatment of MM in Taiwan, allowing us to observe changes in treatment patterns and survival over time.

Limitations of the study include the lack of information on clinical stage, clinical progression, and clinical responses to therapy, which did not allow us to directly link treatment with clinical benefit.

In conclusion, the epidemiology and treatment of MM in Taiwan are changing. The uptake of novel agents for first-line treatment of MM in Taiwan has been high and the use of transplantation continues to increase. High mortality among older adults with MM requires further investigation to understand how treatment can be optimized in this age group. Over the study period, MM case fatality decreased and OS improved, but MM remains an incurable disease. Research to understand the etiologic events underlying the rise in MM in Asia is needed.

## Electronic supplementary material

ESM 1 (DOCX 61.7 kb)

## References

[1] Globocan 2012: Estimated cancer incidence, mortality and prevalence worldwide in 2012. Multiple Myeloma. Available at http://globocan.iarc.fr/Default.aspx Accessed 24 August 2017. .

[2] Cowan AJ, Allen C, Barac A, Basaleem H, Bensenor I, Curado MP, Foreman K, Gupta R, Harvey J, Hosgood HD, Jakovljevic M, Khader Y, Linn S, Lad D, Mantovani L, Nong VM, Mokdad A, Naghavi M, Postma M, Roshandel G, Shackelford K, Sisay M, Nguyen CT, Tran TT, Xuan BT, Ukwaja KN, Vollset SE, Weiderpass E, Libby EN, Fitzmaurice C (2018). Global Burden of Multiple Myeloma: A Systematic Analysis for the Global Burden of Disease Study 2016. JAMA Oncol.

[3] Ries LAG, Eisner MP, Kosary CL (2003). SEER Cancer Statistics Review, 1975-2000.

[4] Landgren O, Weiss BM (2009). Patterns of monoclonal gammopathy of undetermined significance and multiple myeloma in various ethnic/racial groups: support for genetic factors in pathogenesis. Leukemia.

[5] Wu TY, Majeed A, Kuo KN (2010). An overview of the healthcare system in Taiwan. London J Prim Care (Abingdon).

[6] Tang CH, Liu HY, Hou HA, Qiu H, Huang KC, Siggins S, Rothwell LA, Liu Y (2018). Epidemiology of multiple myeloma in Taiwan, a population based study. Cancer Epidemiol.

[7] Huang TC, Chen JH, Wu YY (2019). Burden of Multiple Myeloma in Taiwan. JAMA Oncol.

[8] Chou FH, Tsai KY, Chou YM (2013). The incidence and all-cause mortality of pneumonia in patients with schizophrenia: a nine-year follow-up study. J Psychiatr Res.

[9] Tsai KY, Lee CC, Chou YM, Shen SP, Su CY, Wu HC, Huang MW, Shie JP, Chou FH (2014). The risks of major osteoporotic fractures in patients with schizophrenia: a population-based 10-year follow-up study. Schizophr Res.

[10] Huang TC, Chen JH, Wu YY, Chang PY, Dai MS, Chao TY, Kao WY, Chen YC, Ho CL (2015). The treatment outcome of multiple myeloma patients ineligible for hematopoietic transplantation--a single institutional experience in Taiwan. Ann Hematol.

[11] Kumar SK, Dispenzieri A, Lacy MQ, Gertz MA, Buadi FK, Pandey S, Kapoor P, Dingli D, Hayman SR, Leung N, Lust J, McCurdy A, Russell SJ, Zeldenrust SR, Kyle RA, Rajkumar SV (2014). Continued improvement in survival in multiple myeloma: changes in early mortality and outcomes in older patients. Leukemia.

[12] Wang X, Li Y, Yan X (2016). Efficacy and Safety of Novel Agent-Based Therapies for Multiple Myeloma: A Meta-Analysis. Biomed Res Int.

[13] Kim K, Lee JH, Kim JS, Min CK, Yoon SS, Shimizu K, Chou T, Kosugi H, Suzuki K, Chen W, Hou J, Lu J, Huang XJ, Huang SY, Chng WJ, Tan D, Teoh G, Chim CS, Nawarawong W, Siritanaratkul N, Durie BG (2014). Clinical profiles of multiple myeloma in Asia-An Asian Myeloma Network study. Am J Hematol.

[14] Hungria VTM, Lee JH, Maiolino A, de Queiroz CE, Martinez G, Bittencourt R, Duarte GO, Fantl DB, Navarro JR, Conte G, Gomez-Almaguer D, Ruiz-Arguelles GJ, Kim K, Shimizu K, Chen W, Huang SY, Chng WJ, Chim CS, Nawarawong W, Durie B (2019). Survival differences in multiple myeloma in Latin America and Asia: a comparison involving 3664 patients from regional registries. Ann Hematol.

[15] Ludwig H, Bolejack V, Crowley J, Blade J, Miguel JS, Kyle RA, Rajkumar SV, Shimizu K, Turesson I, Westin J, Sonneveld P, Cavo M, Boccadoro M, Palumbo A, Tosi P, Harousseau JL, Attal M, Barlogie B, Stewart AK, Durie B (2010). Survival and years of life lost in different age cohorts of patients with multiple myeloma. J Clin Oncol.

[16] Cohen YC, Zuckerman T, Yeshurun M, Perez G, Magen H, Henig I, Levi I, Shargian L, Trestman S, Rouvio U, Naparstek E, Ganon-Elazar E, Avivi I, Ram R (2017). Efficacy and safety of autologous hematopoietic cell transplantation in elderly patients with multiple myeloma: a retrospective national multi-site cohort study. Ann Hematol.

[17] Sharma M, Zhang MJ, Zhong X, Abidi MH, Akpek G, Bacher U, Callander NS, Dispenzieri A, Freytes CO, Fung HC, Gale RP, Gasparetto C, Gibson J, Holmberg LA, Kindwall-Keller TL, Klumpp TR, Krishnan AY, Landau HJ, Lazarus HM, Lonial S, Maiolino A, Marks DI, Mehta P, Mikhael Med JR, Nishihori T, Olsson R, Ramanathan M, Roy V, Savani BN, Schouten HC, Scott E, Tay J, Vesole DH, Vogl DT, Hari P, To LB (2014). Older patients with myeloma derive similar benefit from autologous transplantation. Biol Blood Marrow Transplant.

[18] Costa LJ, Brill IK, Omel J, Godby K, Kumar SK, Brown EE (2017). Recent trends in multiple myeloma incidence and survival by age, race, and ethnicity in the United States. Blood Adv.

[19] Perrotta Carla, Staines Anthony, Codd Mary, Kleefeld Silke, Crowley Dominique, T’ Mannetje Andrea, Becker Nicholas, Brennan Paul, De Sanjosé Silvia, Foretova Lenka, Maynadié Marck, Nieters Alexandra, Boffetta Paolo, Cocco Pierluggi (2012). Multiple Myeloma and lifetime occupation: results from the EPILYMPH study. Journal of Occupational Medicine and Toxicology.

[20] Sergentanis TN, Zagouri F, Tsilimidos G, Tsagianni A, Tseliou M, Dimopoulos MA, Psaltopoulou T (2015). Risk Factors for Multiple Myeloma: A Systematic Review of Meta-Analyses. Clin Lymphoma Myeloma Leuk.

[21] Campa Daniele, Martino Alessandro, Macauda Angelica, Dudziński Marek, Suska Anna, Druzd-Sitek Agnieszka, Raab Marc-Steffen, Moreno Victor, Huhn Stefanie, Butrym Aleksandra, Sainz Juan, Szombath Gergely, Rymko Marcin, Marques Herlander, Lesueur Fabienne, Vangsted Annette Juul, Vogel Ulla, Kruszewski Marcin, Subocz Edyta, Buda Gabriele, Iskierka-Jażdżewska Elżbieta, Ríos Rafael, Merz Maximilian, Schöttker Ben, Mazur Grzegorz, Perrial Emeline, Martinez-Lopez Joaquin, Butterbach Katja, García Sanz Ramón, Goldschmidt Hartmut, Brenner Hermann, Jamroziak Krzysztof, Reis Rui Manuel, Kadar Katalin, Dumontet Charles, Wątek Marzena, Haastrup Eva Kannik, Helbig Grzegorz, Jurczyszyn Artur, Jerez Andrés, Varkonyi Judit, Barington Torben, Grzasko Norbert, Zaucha Jan Maciej, Andersen Vibeke, Zawirska Daria, Canzian Federico (2019). Genetic polymorphisms in genes of class switch recombination and multiple myeloma risk and survival: an IMMEnSE study. Leukemia & Lymphoma.

[22] Wei X, Calvo-Vidal MN, Chen S, Wu G, Revuelta MV, Sun J, Zhang J, Walsh MF, Nichols KE, Joseph V, Snyder C, Vachon CM, McKay JD, Wang SP, Jayabalan DS, Jacobs LM, Becirovic D, Waller RG, Artomov M, Viale A, Patel J, Phillip J, Chen-Kiang S, Curtin K, Salama M, Atanackovic D, Niesvizky R, Landgren O, Slager SL, Godley LA, Churpek J, Garber JE, Anderson KC, Daly MJ, Roeder RG, Dumontet C, Lynch HT, Mullighan CG, Camp NJ, Offit K, Klein RJ, Yu H, Cerchietti L, Lipkin SM (2018). Germline Lysine-Specific Demethylase 1 (LSD1/KDM1A) Mutations Confer Susceptibility to Multiple Myeloma. Cancer Res.

[23] Went M, Sud A, Forsti A, Halvarsson BM, Weinhold N, Kimber S, van Duin M, Thorleifsson G, Holroyd A, Johnson DC, Li N, Orlando G, Law PJ, Ali M, Chen B, Mitchell JS, Gudbjartsson DF, Kuiper R, Stephens OW, Bertsch U, Broderick P, Campo C, Bandapalli OR, Einsele H, Gregory WA, Gullberg U, Hillengass J, Hoffmann P, Jackson GH, Jockel KH, Johnsson E, Kristinsson SY, Mellqvist UH, Nahi H, Easton D, Pharoah P, Dunning A, Peto J, Canzian F, Swerdlow A, Eeles RA, Kote-Jarai Z, Muir K, Pashayan N, Nickel J, Nothen MM, Rafnar T, Ross FM, da Silva Filho MI, Thomsen H, Turesson I, Vangsted A, Andersen NF, Waage A, Walker BA, Wihlborg AK, Broyl A, Davies FE, Thorsteinsdottir U, Langer C, Hansson M, Goldschmidt H, Kaiser M, Sonneveld P, Stefansson K, Morgan GJ, Hemminki K, Nilsson B, Houlston RS, consortium P (2018) Identification of multiple risk loci and regulatory mechanisms influencing susceptibility to multiple myeloma. Nat Commun 9 (1):3707. doi:10.1038/s41467-018-04989-w 10.1038/s41467-018-04989-w PMC6137048 30213928

