# Supplementary material for: Treatment evolution and improved survival in multiple myeloma in Taiwan
Source: Ann Hematol. 2019 Dec 5;99(2):321–30. doi: 10.1007/s00277-019-03858-w (PMC6976543; doi:10.1007/s00277-019-03858-w)
Supplement: Supplementary file 1 — (DOCX 61.7 kb) [file 277_2019_3858_MOESM1_ESM.docx]

**SUPPLEMENT**

**Annals of Hematology**

**Treatment evolution and improved survival in multiple myeloma in Taiwan**

Chao-Hsiun Tang, Hsin-An Hou, Kuan-Chih Huang, Hong Qiu, Yanfang Liu

**Table S1** Demographic and clinical characteristics of patients with newly diagnosed multiple myeloma from 2007-2015, Taiwan (N=4387)

|  | **All MM patients** | | **2007-2008** | | **2009-2010** | | **2011-2012** | | **2013-2015** | |
| --- | --- | --- | --- | --- | --- | --- | --- | --- | --- | --- |
| **Total number** | **4387** | | **786** | | **914** | | **1023** | | **1664** | |
| **Variables** | **n** | **%** | **N** | **%** | **n** | **%** | **n** | **%** | **n** | **%** |
| **Gender** |  |  |  |  |  |  |  |  |  |  |
| Male | 2445 | 55.7 | 452 | 57.5 | 536 | 58.6 | 590 | 57.7 | 867 | 52.1 |
| Female | 1942 | 44.3 | 334 | 42.5 | 378 | 41.4 | 433 | 42.3 | 797 | 47.9 |
| **Age, mean (SD)** | 67.6 (12.1) |  | 67.3 (12.3) |  | 67.7 (12.1) |  | 67.7 (12.3) |  | 67.6 (12.0) | |
| <30 | 7 | 0.2 | 1 | 0.1 | 1 | 0.1 | 0 | 0 | 5 | 0.3 |
| 30-39 | 56 | 1.3 | 10 | 1.3 | 17 | 1.9 | 13 | 1.3 | 16 | 1.0 |
| 40-49 | 293 | 6.7 | 66 | 8.4 | 60 | 6.6 | 73 | 7.1 | 94 | 5.6 |
| 50-59 | 818 | 18.6 | 150 | 19.1 | 165 | 18.1 | 206 | 20.1 | 297 | 17.8 |
| 60-69 | 1180 | 26.9 | 195 | 24.8 | 236 | 25.8 | 255 | 24.9 | 494 | 29.7 |
| 70-79 | 1282 | 29.2 | 242 | 30.8 | 289 | 31.6 | 292 | 28.5 | 459 | 27.6 |
| ≧80 | 751 | 17.1 | 122 | 15.5 | 146 | 16.0 | 184 | 18.0 | 299 | 18.0 |
| **Geographical area** |  |  |  |  |  |  |  |  |  |  |
| Taipei | 1469 | 33.5 | 261 | 33.2 | 351 | 38.4 | 328 | 32.1 | 529 | 31.8 |
| Northern | 614 | 14.0 | 118 | 15.0 | 129 | 14.1 | 138 | 13.5 | 229 | 13.8 |
| Central | 871 | 19.9 | 158 | 20.1 | 154 | 16.8 | 230 | 22.5 | 329 | 19.8 |
| Southern | 656 | 15.0 | 115 | 14.6 | 126 | 13.8 | 155 | 15.2 | 260 | 15.6 |
| Kaohsiung & Pingtung | 638 | 14.5 | 107 | 13.6 | 119 | 13.0 | 144 | 14.1 | 268 | 16.1 |
| Eastern | 139 | 3.2 | 27 | 3.4 | 35 | 3.8 | 28 | 2.7 | 49 | 2.9 |
| **Index year** |  |  |  |  |  |  |  |  |  |  |
| 2007 | 383 | 8.7 | 383 | 48.7 | - | - | - | - | - | - |
| 2008 | 403 | 9.2 | 403 | 51.3 | - | - | - | - | - | - |
| 2009 | 435 | 9.9 | - | - | 435 | 47.6 | - | - | - | - |
| 2010 | 479 | 10.9 | - | - | 479 | 52.4 | - | - | - | - |
| 2011 | 497 | 11.3 | - | - | - | - | 497 | 48.6 | - | - |
| 2012 | 526 | 12.0 | - | - | - | - | 526 | 51.4 | - | - |
| 2013 | 529 | 12.1 | - | - | - | - | - | - | 529 | 31.8 |
| 2014 | 540 | 12.3 | - | - | - | - | - | - | 540 | 32.5 |
| 2015 | 595 | 13.6 | - | - | - | - | - | - | 595 | 35.8 |
| **Comorbidities associated with MM** |  |  |  |  |  |  |  |  |  |  |
| Renal impairment | 774 | 17.6 | 113 | 14.4 | 147 | 16.1 | 187 | 18.3 | 327 | 19.7 |
| Anemia | 1589 | 36.2 | 287 | 36.5 | 319 | 34.9 | 354 | 34.6 | 629 | 37.8 |
| Bone fracture | 788 | 18.0 | 138 | 17.6 | 169 | 18.5 | 183 | 17.9 | 298 | 17.9 |
| Pneumonia | 722 | 16.5 | 136 | 17.3 | 143 | 15.6 | 192 | 18.8 | 251 | 15.1 |
| **Frequency of Transplant** |  |  |  |  |  |  |  |  |  |  |
| 0 | 3853 | 87.8 | 724 | 92.1 | 819 | 89.6 | 887 | 86.7 | 1423 | 85.5 |
| 1 | 499 | 11.4 | 55 | 7.0 | 85 | 9.3 | 127 | 12.4 | 232 | 13.9 |
| ≥2 | 35 | 0.8 | 7 | 0.9 | 10 | 1.1 | 9 | 0.9 | 9 | 0.5 |
| **CCI Deyo, mean (SD)** | 1.9 (2.1) |  | 1.7 (1.9) |  | 1.9 (2.0) |  | 1.8 (2.0) |  | 1.9 (2.3) |  |
| 0 | 1397 | 31.8 | 263 | 33.5 | 267 | 29.2 | 304 | 29.7 | 563 | 33.8 |
| 1 | 951 | 21.7 | 161 | 20.5 | 205 | 22.4 | 248 | 24.2 | 337 | 20.3 |
| 2 | 839 | 19.1 | 161 | 20.5 | 178 | 19.5 | 196 | 19.2 | 304 | 18.3 |
| ≥ 3 | 1200 | 27.4 | 201 | 25.6 | 264 | 28.9 | 275 | 26.9 | 460 | 27.6 |

n, % = number and percentage of patients with the indicated characteristic, SD = standard deviation, CCI = Charlson comorbidity index

**Table S2** Sex-specific and age-specific incidence of multiple myeloma in Taiwan (age-standardized rates per 100,000 population with 95% confidence interval) 2007-2015†

|  | **2007-2008** | | | **2009-2010** | | | **2011-2012** | | | **2013-2015** | | |
| --- | --- | --- | --- | --- | --- | --- | --- | --- | --- | --- | --- | --- |
| **Age (years)** | **Both sexes** | **Male** | **Female** | **Both sexes** | **Male** | **Female** | **Both sexes** | **Male** | **Female** | **Both sexes** | **Male** | **Female** |
| Total | 1.78  (1.61-1.96) | 2.07  (1.81-2.36) | 1.49  (1.28-1.74) | 2.02  (1.84-2.22) | 2.41  (2.13-2.71) | 1.65  (1.42-1.90) | 2.21  (2.03-2.41) | 2.59  (2.31-2.91) | 1.84  (1.61-2.11) | 2.34  (2.15-2.54) | 2.52  (2.24-2.83) | 2.29  (2.03-2.59) |
| <30 | 0.01  (0-0.05) | 0.01  (0-0.11) | 0  (0-0.08) | 0.01  (0-0.05) | 0.01  (0-0.11) | 0  (0-0.09) | 0  (0-0.04) | 0  (0-0.09) | 0  (0-0.09) | 0.02  (0-0.08) | 0.03  (0-0.15) | 0.01  (0-0.11) |
| 30-39 | 0.14  (0.04-0.32) | 0.11  (0.02-0.40) | 0.16  (0.04-0.46) | 0.22  (0.10-0.43) | 0.27  (0.09-0.63) | 0.18  (0.04-0.48) | 0.16  (0.07-0.34) | 0.29  (0.10-0.64) | 0.05  (0.01-0.26) | 0.13  (0.05-0.29) | 0.09  (0.02-0.34) | 0.19  (0.06-0.49) |
| 40-49 | 0.91  (0.63-1.28) | 0.94  (0.55-1.51) | 0.88  (0.50-1.42) | 0.81  (0.55-1.16) | 1.07  (0.65-1.65) | 0.56  (0.28-1.01) | 0.98  (0.69-1.36) | 1.04  (0.62-1.62) | 0.93  (0.55-1.48) | 0.85  (0.58-1.21) | 0.77  (0.42-1.3) | 0.98  (0.58-1.55) |
| 50-59 | 2.56  (2.02-3.21) | 2.81  (2.01-3.82) | 2.33  (1.62-3.24) | 2.58  (2.05-3.20) | 2.98  (2.19-3.97) | 2.19  (1.53-3.04) | 3.05  (2.49-3.70) | 3.40  (2.57-4.41) | 2.72  (1.99-3.62) | 2.8  (2.28-3.41) | 3.00  (2.24-3.94) | 2.63  (1.94-3.5) |
| 60-69 | 6.45  (5.23-7.86) | 7.25  (5.42-9.49) | 5.71  (4.17-7.64) | 7.04  (5.83-8.44) | 7.98  (6.15-10.19) | 6.17  (4.63-8.06) | 6.63  (5.53-7.88) | 7.76  (6.07-9.76) | 5.57  (4.20-7.25) | 7.15  (6.1-8.34) | 7.98  (6.4-9.83) | 6.41  (5.06-8.03) |
| 70-79 | 11.2  (9.3-13.39) | 14.06  (11.05-17.65) | 8.50  (6.25-11.3) | 12.9  (10.88-15.18) | 15.62  (12.43-19.39) | 10.47  (8.05-13.41) | 12.47  (10.53-14.67) | 15.3  (12.17-18.98) | 10.1  (7.78-12.89) | 12.38  (10.5-14.51) | 13.75  (10.86-17.17) | 11.27  (8.89-14.1) |
| ≥80 | 11.99  (9.17-15.4) | 15.44  (11.03-21.06) | 8.51  (5.3-12.95) | 12.66  (9.92-15.92) | 17.18  (12.73-22.68) | 8.15  (5.2-12.17) | 14.33  (11.55-17.58) | 19.07  (14.56-24.55) | 9.79  (6.69-13.81) | 13.71  (11.15-16.68) | 16.06  (12.11-20.91) | 11.6  (8.46-15.52) |

†data from 2007-2012 previously published Tang et al, 2018 [[1](#_ENREF_1)]

**Table S3** Sex-specific and age-specific prevalence of multiple myeloma in Taiwan (age-standardized rates per 100,000 population with 95% confidence interval) 2007-2015†

|  | **2007-2008** | | | **2009-2010** | | | **2011-2012** | | | **2013-2015** | | |
| --- | --- | --- | --- | --- | --- | --- | --- | --- | --- | --- | --- | --- |
| **Age (years)** | **Both sexes** | **Male** | **Female** | **Both sexes** | **Male** | **Female** | **Both sexes** | **Male** | **Female** | **Both sexes** | **Male** | **Female** |
| Total | 5.82  (5.51-6.15) | 6.86  (6.38-7.37) | 4.81  (4.41-5.23) | 6.56  (6.23-6.90) | 7.76  (7.25-8.30) | 5.38  (4.97-5.83) | 7.78  (7.43-8.15) | 8.84  (8.30-9.40) | 6.76  (6.29-7.24) | 8.8  (8.43-9.19) | 9.8  (9.24-10.39) | 8.31  (7.79-8.85) |
| <30 | 0.13  (0.06-0.22) | 0.15  (0.06-0.31) | 0.10  (0.03-0.25) | 0.11  (0.05-0.21) | 0.16  (0.07-0.34) | 0.06  (0.01-0.19) | 0.05  (0.02-0.13) | 0.08  (0.02-0.23) | 0.02  (0-0.13) | 0.12  (0.06-0.23) | 0.08  (0.02-0.23) | 0.03  (0-0.15) |
| 30-39 | 0.64  (0.41-0.96) | 0.81  (0.45-1.35) | 0.48  (0.22-0.91) | 0.73  (0.49-1.06) | 0.75  (0.41-1.26) | 0.71  (0.39-1.19) | 0.81  (0.56-1.15) | 1.04  (0.63-1.60) | 0.61  (0.32-1.05) | 0.51  (0.32-0.78) | 0.56  (0.28-1.01) | 0.53  (0.26-0.96) |
| 40-49 | 3.87  (3.26-4.56) | 4.76  (3.81-5.88) | 2.98  (2.25-3.89) | 3.82  (3.22-4.51) | 4.84  (3.88-5.95) | 2.82  (2.11-3.69) | 4.45  (3.80-5.18) | 5.07  (4.09-6.22) | 3.84  (3.00-4.84) | 2.9  (2.37-3.5) | 3.07  (2.31-4) | 2.88  (2.15-3.77) |
| 50-59 | 9.94  (8.83-11.15) | 10.86  (9.23-12.7) | 9.05  (7.59-10.72) | 10.4  (9.31-11.58) | 11.49  (9.88-13.29) | 9.34  (7.91-10.95) | 12.17  (11.02-13.41) | 13.05  (11.37-14.91) | 11.31  (9.77-13.02) | 10.73  (9.68-11.87) | 11.96  (10.38-13.7) | 9.66  (8.27-11.21) |
| 60-69 | 22.61  (20.27-25.14) | 26.99  (23.35-31.05) | 18.56  (15.68-21.83) | 23.89  (21.61-26.35) | 28.00  (24.46-31.9) | 20.07  (17.20-23.29) | 25.46  (23.25-27.82) | 29.07  (25.71-32.75) | 22.08  (19.26-25.2) | 26.62  (24.55-28.81) | 29.27  (26.17-32.64) | 24.26  (21.54-27.23) |
| 70-79 | 32.64  (29.32-36.23) | 40.17  (34.94-45.96) | 25.48  (21.44-30.05) | 36.51  (33.06-40.23) | 46.00  (40.38-52.18) | 28.13  (24.04-32.73) | 41.01  (37.42-44.85) | 49.83  (44.03-56.18) | 33.57  (29.21-38.39) | 47.16  (43.41-51.15) | 54.08  (48.17-60.51) | 41.48  (36.76-46.65) |
| ≥80 | 24.73  (20.59-29.46) | 30.43  (24.04-38.01) | 19.00  (14.01-25.19) | 28.06  (23.90-32.73) | 36.92  (30.24-44.64) | 19.21  (14.49-24.98) | 33.55  (29.22-38.34) | 42.75  (35.84-50.61) | 24.73  (19.64-30.74) | 51.69  (46.6-57.18) | 63.98  (55.78-73.06) | 40.81  (34.71-47.68) |

†data from 2007-2012 previously published Tang et al, 2018 [[1](#_ENREF_1)]

**Table S4** Sex-specific and age-specific call-cause mortality from multiple myeloma in Taiwan (age-standardized rates per 100,000 population with 95% confidence interval) 2007-2015†

|  | **2007-2008** | | | **2009-2010** | | | **2011-2012** | | | **2013-2015** | | |
| --- | --- | --- | --- | --- | --- | --- | --- | --- | --- | --- | --- | --- |
| **Age (years)** | **Both sexes** | **Male** | **Female** | **Both sexes** | **Male** | **Female** | **Both sexes** | **Male** | **Female** | **Both sexes** | **Male** | **Female** |
| Total | 1.42  (1.27-1.59) | 1.75  (1.51-2.01) | 1.1  (0.92-1.32) | 1.46  (1.31-1.62) | 1.79  (1.55-2.06) | 1.14  (0.95-1.35) | 1.53  (1.37-1.7) | 1.88  (1.63-2.15) | 1.19  (1.00-1.40) | 1.72  (1.56-1.9) | 2.13  (1.87-2.42) | 1.42  (1.21-1.65) |
| <30 | 0.01  (0-0.05) | 0  (0-0.08) | 0.01  (0-0.11) | 0  (0-0.04) | 0  (0-0.09) | 0  (0-0.09) | 0  (0-0.04) | 0  (0-0.09) | 0  (0-0.09) | 0.02  (0-0.08) | 0.02  (0-0.14) | 0.01  (0-0.11) |
| 30-39 | 0.05  (0.01-0.20) | 0.08  (0.01-0.36) | 0.03  (0-0.25) | 0.09  (0.02-0.25) | 0.11  (0.02-0.39) | 0.08  (0.01-0.32) | 0.11  (0.04-0.27) | 0.16  (0.03-0.45) | 0.07  (0.01-0.31) | 0.03  (0-0.15) | 0.07  (0.01-0.32) | 0  (0-0.19) |
| 40-49 | 0.67  (0.43-1.00) | 0.78  (0.42-1.30) | 0.58  (0.28-1.04) | 0.49  (0.29-0.77) | 0.74  (0.40-1.25) | 0.24  (0.07-0.59) | 0.50  (0.30-0.78) | 0.65  (0.34-1.14) | 0.35  (0.13-0.73) | 0.3  (0.15-0.54) | 0.45  (0.2-0.89) | 0.17  (0.04-0.48) |
| 50-59 | 1.71  (1.27-2.25) | 1.87  (1.24-2.72) | 1.55  (0.98-2.32) | 1.58  (1.18-2.08) | 1.84  (1.23-2.64) | 1.33  (0.83-2.02) | 1.54  (1.15-2.02) | 1.80  (1.22-2.57) | 1.29  (0.81-1.95) | 1.43  (1.07-1.89) | 1.86  (1.28-2.63) | 1.03  (0.61-1.62) |
| 60-69 | 5.34  (4.25-6.64) | 6.58  (4.86-8.72) | 4.20  (2.90-5.90) | 4.66  (3.69-5.82) | 5.38  (3.90-7.24) | 3.99  (2.78-5.57) | 4.74  (3.81-5.81) | 5.83  (4.38-7.60) | 3.72  (2.62-5.13) | 3.95  (3.18-4.85) | 4.77  (3.57-6.25) | 3.19  (2.26-4.39) |
| 70-79 | 9.49  (7.74-11.52) | 12.9  (10.02-16.36) | 6.22  (4.33-8.67) | 9.88  (8.12-11.9) | 12.29  (9.47-15.67) | 7.75  (5.68-10.33) | 9.78  (8.07-11.75) | 12.78  (9.94-16.18) | 7.26  (5.31-9.68) | 9.94  (8.27-11.86) | 12.17  (9.47-15.41) | 8.1  (6.1-10.55) |
| ≥80 | 10.63  (7.98-13.87) | 12.78  (8.77-17.99) | 8.46  (5.28-12.87) | 11.94  (9.29-15.12) | 16.27  (11.95-21.63) | 7.64  (4.79-11.56) | 11.28  (8.84-14.19) | 14.62  (10.71-19.5) | 8.06  (5.30-11.77) | 17.88  (14.95-21.23) | 23.59  (18.73-29.32) | 12.82  (9.53-16.9) |

†data from 2007-2012 previously published Tang et al, 2018 [[1](#_ENREF_1)]

**Table S5** Number (percentage) of patients using first line treatment regimens: 1969 patients in 2007 to 2012 and 1576 patients in 2013 to 2015

| **First line treatment** | **All patients**  **N=1969** | **2007**  **N=273** | **2008**  **N=288** | **2009**  **n=321** | **2010**  **N=321** | **2011**  **N=370** | **2012**  **N=396** | **2013**  **N=505** | **2014**  **N=506** | **2015**  **n=565** |
| --- | --- | --- | --- | --- | --- | --- | --- | --- | --- | --- |
| Steroid | 338 (17.2) | 79 (28.9) | 101 (35.1) | 62 (19.3) | 43 (13.4) | 29 (7.8) | 24 (6.1) | 54 (10.7) | 69 (13.6) | 55 (9.7) |
| Chemotherapy | 557 (28.3) | 193 (70.7) | 180 (62.5) | 109 (34) | 27 (8.4) | 37 (10) | 11 (2.8) | 24 (4.8) | 6 (1.2) | 5 (0.9) |
| Novel + Chemotherapy | 506 (25.7) | 1 (0.4) | 5 (1.7) | 97 (30.2) | 140 (43.6) | 137 (37) | 126 (31.8) | 113 (22.4) | 84 (16.6) | 72 (12.7) |
| Novel agent | 568 (28.8) | 0 (0) | 2 (0.7) | 53 (16.5) | 111 (34.6) | 167 (45.1) | 235 (59.3) | 314 (62.2) | 347 (68.6) | 433 (76.6) |

**Reference**

1. Tang CH, Liu HY, Hou HA, Qiu H, Huang KC, Siggins S, Rothwell LA, Liu Y (2018) Epidemiology of multiple myeloma in Taiwan, a population based study. Cancer Epidemiol 55:136-141. doi:10.1016/j.canep.2018.06.003
